# Supplementary material for: Imbalanced machine learning classification models for removal biosimilar drugs and increased activity in patients with rheumatic diseases
Source: PLoS One. 2023 Nov 30;18(11):e0291891. doi: 10.1371/journal.pone.0291891 (PMC10688843; doi:10.1371/journal.pone.0291891)
Supplement: S1 File — (PDF) [file pone.0291891.s001.pdf]

```
## Data analysis of variable 'DIF', worsening of patients. First, we do a mutual information test
##, and then we compute different imbalanced machine learning methods


import pandas as pd

df = pd.read_excel('datos.xlsx')

df=df.dropna()

X = df.drop(['DIF'], axis=1)

y = df['DIF']

activity_count = y.value_counts()

activity_count

y.value_counts().plot.pie(autopct='%.2f')

print(df.columns)


###Mutual information code

import pandas as pd

import numpy as np

from sklearn.feature_selection import mutual_info_regression

import matplotlib.pyplot as plt

target_variable = 'DIF'

feature_variables = ['SEX', 'AGE', 'DIAGNOSIS', 'IMMUNOLOGY', 'FAME ', 'CORTICOIDS',

    'BIOLOGICAL', 'BIOSIMILAR ', 'ELEVATED_INITIAL_VSG', 'INITIAL_VSG',

    'ELEVATED_INITIAL_PCR', 'INITIAL_PCR', 'ELEVATED_FINAL_VSG',

    'FINAL_VSG', 'ELEVATED_FINAL_PCR',

    'SWITCHING', 'CHANGE_BIOSIMILAR', 'OPTIMIZATION','ACTIVITY_16weeks']


# Number of repetitions

n_repeats = 1000


# List to store the mutual information values

mutual_info_values = []
```

```
# Repeat the experiment n_repeats times
for _ in range(n_repeats):
    # Calcular la información mutua de la variable objetivo con respecto al resto de variables
    mutual_info = mutual_info_regression(df[feature_variables], df[target_variable])
    mutual_info_values.append(mutual_info)

# Convert the list to a NumPy array for easier calculation
mutual_info_values = np.array(mutual_info_values)

# Calculate the mean of the mutual information values
mean_mutual_info = mutual_info_values.mean(axis=0)
mutual_info_df = pd.DataFrame({
    'Variable': feature_variables,
    'Average Mutual Information': mean_mutual_info
})

# Display the DataFrame with the average mutual information
print(mutual_info_df)

# plot the mutual information test
plt.figure(figsize=(8, 6))
plt.bar(mutual_info_df['Variable'], mutual_info_df['Average Mutual Information'])  # Use
'Average Mutual Information' here
plt.xlabel('Variables')
plt.ylabel('Mutual Information')
plt.title('Importance of variables explaining WORSENING')
plt.xticks(rotation=90)
plt.tight_layout()
plt.savefig('mutualinfo11.jpeg', format='jpeg', dpi=300)
plt.show()
print(mutual_info_df)
```

```
#upload LIBRARIES
from numpy import mean
from sklearn.datasets import make_classification
from sklearn.model_selection import cross_val_score
from sklearn.model_selection import RepeatedStratifiedKFold
from sklearn.tree import DecisionTreeClassifier
import pandas as pd
import numpy as np
import matplotlib.pyplot as plt
import seaborn as sns

from sklearn.metrics import confusion_matrix
from sklearn.metrics import classification_report
from sklearn.model_selection import train_test_split
from sklearn.linear_model import LogisticRegression
from sklearn.decomposition import PCA
from sklearn.tree import DecisionTreeClassifier
import pandas as pd
import numpy as np
import matplotlib.pyplot as plt
import seaborn as sns

from sklearn.metrics import confusion_matrix
from sklearn.metrics import classification_report
from sklearn.model_selection import train_test_split
from sklearn.linear_model import LogisticRegression
from sklearn.decomposition import PCA
from sklearn.tree import DecisionTreeClassifier

from pylab import rcParams
```

```
from imblearn.under_sampling import NearMiss
from imblearn.over_sampling import RandomOverSampler
from imblearn.combine import SMOTETomek
from imblearn.ensemble import BalancedBaggingClassifier
from numpy import mean
from sklearn.datasets import make_classification
from sklearn.model_selection import cross_val_score
from sklearn.model_selection import RepeatedStratifiedKFold
from sklearn.tree import DecisionTreeClassifier

from collections import Counter
import scikitplot as skplt
import matplotlib.pyplot as plt

from pylab import rcParams

from imblearn.under_sampling import NearMiss
from imblearn.over_sampling import RandomOverSampler
from imblearn.combine import SMOTETomek
from imblearn.ensemble import BalancedBaggingClassifier

from collections import Counter
#Function to show results
def mostrar_resultados(y_test, pred_y):
    conf_matrix = confusion_matrix(y_test, pred_y)
    plt.figure(figsize=(8, 8))
    sns.heatmap(conf_matrix, annot=True, fmt="d");
    plt.title("Confusion matrix")
    plt.ylabel('True class')
    plt.xlabel('Predicted class')
    plt.show()
```

```
    print (classification_report(y_test, pred_y))
y=df['DIF']
X=df[['AGE','SWITCHING','ACTIVITY_16weeks','ELEVATED_INITIAL_PCR', 'BIOLOGICAL']]
X_train, X_test, y_train, y_test = train_test_split(X, y, train_size=0.7, stratify=y)

##ROS

def run_model_balanced(X_train, X_test, y_train, y_test):
    rus = RandomUnderSampler(sampling_strategy=1)
    X_train_resampled, y_train_resampled = rus.fit_resample(X_train, y_train)

    tr = tree.DecisionTreeClassifier(max_leaf_nodes=4, random_state=0)

    model = tr.fit(X_train_resampled, y_train_resampled)
    return model

model = run_model_balanced(X_train, X_test, y_train, y_test)
pred_y = model.predict(X_test)
mostrar_resultados(y_test, pred_y)

scores = cross_val_score(model, X_test, y_test, scoring='roc_auc', n_jobs=-1)

mean_roc_auc = np.mean(scores)
print('Mean ROC AUC: %.3f' % mean_roc_auc)
y_pred = model.predict_proba(X_test)[:,1]

from plot_metric.functions import BinaryClassification
# Visualisation with plot_metric
bc = BinaryClassification(y_test, y_pred, labels=["Class 0", "Class 1"])

# Figures
```

```
plt.figure(figsize=(6,6))

bc.plot_roc_curve()

plt.show()

# Get the list of feature names

feature_names = list(X_train.columns)


# Plot the decision tree with feature names

plt.figure(figsize=(15, 10))

tree.plot_tree(model, feature_names=feature_names, class_names=["Class 0", "Class 1"],
filled=True)

plt.show()



##ROS


y=df['DIF']

X=df[['AGE','SWITCHING','ACTIVITY_16weeks','ELEVATED_INITIAL_PCR', 'BIOLOGICAL']]

X_train, X_test, y_train, y_test = train_test_split(X, y, train_size=0.7, stratify=y)

from imblearn.over_sampling import RandomOverSampler

from sklearn import tree

from sklearn.model_selection import cross_val_score

import numpy as np


def run_model_balanced(X_train, X_test, y_train, y_test):

   ros = RandomOverSampler(sampling_strategy=1)

   X_train_resampled, y_train_resampled = ros.fit_resample(X_train, y_train)


   tr = tree.DecisionTreeClassifier(max_leaf_nodes=4, random_state=0)


   model = tr.fit(X_train_resampled, y_train_resampled)

   return model
```

```
model = run_model_balanced(X_train, X_test, y_train, y_test)
pred_y = model.predict(X_test)
mostrar_resultados(y_test, pred_y)
scores = cross_val_score(model, X_test, y_test, scoring='roc_auc', n_jobs=-1)
mean_roc_auc = np.mean(scores)
print('Mean ROC AUC: %.3f' % mean_roc_auc)
feature_names = list(X_train.columns)

# Plot the decision tree with feature names
plt.figure(figsize=(15, 10))
tree.plot_tree(model, feature_names=feature_names, class_names=["Class 0", "Class 1"],
filled=True)
plt.show()
# Use predict_proba to predict probability of the class
y_pred = model.predict_proba(X_test)[:,1]

from plot_metric.functions import BinaryClassification
# Visualisation with plot_metric
bc = BinaryClassification(y_test, y_pred, labels=["Class 0", "Class 1"])

# Figures
plt.figure(figsize=(5,5))
bc.plot_roc_curve()
plt.show()

## SMOTE model
y=df['DIF']
X=df[['AGE','SWITCHING','ACTIVITY_16weeks','ELEVATED_INITIAL_PCR', 'BIOLOGICAL']]
X_train, X_test, y_train, y_test = train_test_split(X, y, train_size=0.7, stratify=y)
def run_model_balanced(X_train, X_test, y_train, y_test):
```

```
    smote = SMOTE(sampling_strategy=0.7)
    X_train_resampled, y_train_resampled = smote.fit_resample(X_train, y_train)
    tr = tree.DecisionTreeClassifier(max_leaf_nodes=4, random_state=0)

    model = tr.fit(X_train_resampled, y_train_resampled)
    return model

model = run_model_balanced(X_train, X_test, y_train, y_test)
pred_y = model.predict(X_test)
mostrar_resultados(y_test, pred_y)
scores = cross_val_score(model, X_test, y_test, scoring='roc_auc', n_jobs=-1)
mean_roc_auc = np.mean(scores)

print('Mean ROC AUC: %.3f' % mean_roc_auc)
y_pred = model.predict_proba(X_test)[:,1]

from plot_metric.functions import BinaryClassification
# Visualisation with plot_metric
bc = BinaryClassification(y_test, y_pred, labels=["Class 0", "Class 1"])

# Figures
plt.figure(figsize=(5,5))
bc.plot_roc_curve()
plt.show()
# Get the list of feature names
feature_names = list(X_train.columns)

# Plot the decision tree with feature names
plt.figure(figsize=(15, 10))
tree.plot_tree(model, feature_names=feature_names, class_names=["Class 0", "Class 1"],
filled=True)
```

```
plt.show()


##SMOTE Tomek
y=df['DIF']
X=df[['AGE','SWITCHING','ACTIVITY_16weeks','ELEVATED_INITIAL_PCR', 'BIOLOGICAL']]
X_train, X_test, y_train, y_test = train_test_split(X, y, train_size=0.7, stratify=y)
from imblearn.combine import SMOTETomek


def run_model_balanced(X_train, X_test, y_train, y_test):
   smote = SMOTETomek(sampling_strategy=0.7)
   X_train_resampled, y_train_resampled = smote.fit_resample(X_train, y_train)
   tr = tree.DecisionTreeClassifier(max_leaf_nodes=4, random_state=0)
   model = tr.fit(X_train_resampled, y_train_resampled)
   return model


model = run_model_balanced(X_train, X_test, y_train, y_test)
pred_y = model.predict(X_test)
mostrar_resultados(y_test, pred_y)


scores = cross_val_score(model, X_test, y_test, scoring='roc_auc', n_jobs=-1)
mean_roc_auc = np.mean(scores)
print('Mean ROC AUC: %.3f' % mean_roc_auc)


# Use predict_proba to predict probability of the class
y_pred = model.predict_proba(X_test)[:,1]


from plot_metric.functions import BinaryClassification
# Visualisation with plot_metric
bc = BinaryClassification(y_test, y_pred, labels=["Class 0", "Class 1"])


# Figures
```

```
plt.figure(figsize=(5,5))

bc.plot_roc_curve()

plt.show()

# Get the list of feature names

feature_names = list(X_train.columns)


# Plot the decision tree with feature names

plt.figure(figsize=(15, 10))

tree.plot_tree(model, feature_names=feature_names, class_names=["Class 0", "Class 1"],
filled=True)

plt.show()




## Imbalanced logistic regression

y=df['DIF']

X=df[['AGE','ACTIVITY_16weeks']]


X_train, X_test, y_train, y_test = train_test_split(X, y, train_size=0.7, stratify=y)

def run_model_balanced(X_train, X_test, y_train, y_test):

clf = LogisticRegression(C=1.0,penalty='l2',random_state=1,solver="newton-
cg",class_weight={0: 1,1: 10})

    clf.fit(X_train, y_train)

    return clf


model = run_model_balanced(X_train, X_test, y_train, y_test)

pred_y = model.predict(X_test)

mostrar_resultados(y_test, pred_y)


# evaluate model

scores = cross_val_score(model, X_test, y_test, scoring='roc_auc', n_jobs=-1)

# summarize performance

print('Mean ROC AUC: %.3f' % mean(scores))
```

```
# Use predict_proba to predict probability of the class
y_pred = model.predict_proba(X_test)[:,1]

from plot_metric.functions import BinaryClassification
# Visualisation with plot_metric
bc = BinaryClassification(y_test, y_pred, labels=["Class 0", "Class 1"])

# Figures
plt.figure(figsize=(6,6))
bc.plot_roc_curve()
plt.show()
import statsmodels.api as sm

# Fit a logistic regression model using statsmodels to get p-values
X_train_stats = sm.add_constant(X_train)  # Add constant for the intercept term
logit_model = sm.Logit(y_train, X_train_stats)
result = logit_model.fit()

# Print p-values
print("\nP-values:")
print(result.pvalues)

# Write the explicit logistic regression model
print("\nLogistic Regression Model:")
print("y = 1 / (1 + e^(-(", end='')
for i, coef in enumerate(result.params):
    if i == 0:
        print(f"{coef:.4f}", end='')
    else:
        print(f" + {coef:.4f} * {X_train.columns[i-1]}", end='')
print("))")
```

```
## Balanced bagging classifier
y=df['DIF']
X=df[['AGE','SWITCHING','ACTIVITY_16weeks','ELEVATED_INITIAL_PCR', 'BIOLOGICAL']]
X_train, X_test, y_train, y_test = train_test_split(X, y, train_size=0.7, stratify=y)
def run_model_balanced(X_train, X_test, y_train, y_test):
    bbc = BalancedBaggingClassifier(base_estimator=DecisionTreeClassifier(),
                        sampling_strategy=1,
                        replacement=False,
                        random_state=0)
    bbc.fit(X_train, y_train)
    return bbc

model = run_model_balanced(X_train, X_test, y_train, y_test)
pred_y = model.predict(X_test)
mostrar_resultados(y_test, pred_y)

# evaluate model
scores = cross_val_score(model, X_test, y_test, scoring='roc_auc', n_jobs=-1)
# summarize performance
print('Mean ROC AUC: %.3f' % mean(scores))
# Use predict_proba to predict probability of the class
y_pred = model.predict_proba(X_test)[:,1]

from plot_metric.functions import BinaryClassification
# Visualisation with plot_metric
bc = BinaryClassification(y_test, y_pred, labels=["Class 0", "Class 1"])

# Figures
plt.figure(figsize=(6,6))
bc.plot_roc_curve()
```

```
plt.show()

#XGBOOST
y=df['DIF']
X=df[['AGE','SWITCHING','ACTIVITY_16weeks','ELEVATED_INITIAL_PCR', 'BIOLOGICAL']]
X_train, X_test, y_train, y_test = train_test_split(X, y, train_size=0.7, stratify=y)
from xgboost import XGBClassifier
def run_model_balanced(X_train, X_test, y_train, y_test):
    xgb = XGBClassifier(scale_pos_weight=1000)
    xgb=xgb.fit(X_train,y_train)
    return xgb

model = run_model_balanced(X_train, X_test, y_train, y_test)
pred_y = model.predict(X_test)
mostrar_resultados(y_test, pred_y)

# evaluate model
scores = cross_val_score(model, X_test, y_test, scoring='roc_auc', n_jobs=-1)
# summarize performance
print('Mean ROC AUC: %.3f' % mean(scores))

# Use predict_proba to predict probability of the class
y_pred = model.predict_proba(X_test)[:,1]

from plot_metric.functions import BinaryClassification
# Visualisation with plot_metric
bc = BinaryClassification(y_test, y_pred, labels=["Class 0", "Class 1"])

# Figures
plt.figure(figsize=(6,6))
bc.plot_roc_curve()
```

```
plt.show()

## Balanced Random Forest Classifier
y=df['DIF']
X=df[['AGE','SWITCHING','ACTIVITY_16weeks','ELEVATED_INITIAL_PCR', 'BIOLOGICAL']]
X_train, X_test, y_train, y_test = train_test_split(X, y, train_size=0.7, stratify=y)
from imblearn.ensemble import BalancedRandomForestClassifier
def run_model_balanced(X_train, X_test, y_train, y_test):
   brf = BalancedRandomForestClassifier(n_estimators=500,class_weight={0:30,1:1})
   brf=brf.fit(X_train,y_train)
   return brf

model = run_model_balanced(X_train, X_test, y_train, y_test)
pred_y = model.predict(X_test)
mostrar_resultados(y_test, pred_y)

# evaluate model
scores = cross_val_score(model, X_test, y_test, scoring='roc_auc', n_jobs=-1)
# summarize performance
print('Mean ROC AUC: %.3f' % mean(scores))
# Use predict_proba to predict probability of the class
y_pred = model.predict_proba(X_test)[:,1]

from plot_metric.functions import BinaryClassification
# Visualisation with plot_metric
bc = BinaryClassification(y_test, y_pred, labels=["Class 0", "Class 1"])

# Figures
plt.figure(figsize=(5,5))
bc.plot_roc_curve()
plt.show()
```

```
##ADASYN
y=df['DIF']
X=df[['AGE','SWITCHING','ACTIVITY_16weeks','ELEVATED_INITIAL_PCR', 'BIOLOGICAL']]
X_train, X_test, y_train, y_test = train_test_split(X, y, train_size=0.7, stratify=y)
import pandas as pd
from sklearn.model_selection import train_test_split, cross_val_score
from sklearn.tree import DecisionTreeClassifier
from imblearn.over_sampling import ADASYN
from numpy import mean

def run_adasyn(X_train, X_test, y_train, y_test):
   adasyn = ADASYN(sampling_strategy=1, random_state=0)
   X_train_resampled, y_train_resampled = adasyn.fit_resample(X_train, y_train)
   model = DecisionTreeClassifier(random_state=0, class_weight={0: 1, 1: 1000})
   model.fit(X_train_resampled, y_train_resampled)
   return model
model = run_adasyn(X_train, X_test, y_train, y_test)
pred_y = model.predict(X_test)
mostrar_resultados(y_test, pred_y)

# Evaluate the model using cross-validation
scores = cross_val_score(model, X_test, y_test, scoring='roc_auc', cv=5, n_jobs=-1)

# Summarize performance
print('Mean ROC AUC: %.3f' % mean(scores))
from plot_metric.functions import BinaryClassification
# Visualisation with plot_metric
bc = BinaryClassification(y_test, pred_y, labels=["Class 0", "Class 1"])

# Figures
```

```
plt.figure(figsize=(5,5))
bc.plot_roc_curve()
plt.show()
```
